# Enhanced diabetic wound healing using platelet-derived extracellular vesicles and reduced graphene oxide in polymer-coordinated hydrogels

**DOI:** 10.1186/s12951-023-02068-x

**Published:** 2023-09-04

**Authors:** Ping-Chien Hao, Thierry Burnouf, Chih-Wei Chiang, Pei-Ru Jheng, Sabine Szunerits, Jen-Chang Yang, Er-Yuan Chuang

**Affiliations:** 1 Graduate Institute of Biomedical Materials and Tissue Engineering, College of Biomedical Engineering, Taipei Medical University, Taipei, 11031 Taiwan; 2 International Ph.D. Program in Biomedical Engineering, College of Biomedical Engineering, Taipei Medical University, Taipei, 11031 Taiwan; 3 Graduate Institute of Biomedical Electronics and Bioinformatics, National Taiwan University, Taipei, 10617 Taiwan; 4 Department of Orthopedics, Taipei Medical University Hospital, Taipei, 11031 Taiwan; 5 Univ. Lille, CNRS, Centrale Lille, Univ. Polytechnique Hauts-de-France, UMR 8520, IEMN, Lille, F- 59000 France; 6 Graduate Institute of Nanomedicine and Medical Engineering, College of Biomedical Engineering, Taipei Medical University, Taipei, 110-52 Taiwan; 7 Cell Physiology and Molecular Image Research Center, Taipei Medical University-Wan Fang Hospital, Taipei, 11696 Taiwan; 8 Precision Medicine and Translational Cancer Research Center, Taipei Medical University Hospital, Taipei, 11031 Taiwan

**Keywords:** Hydrogel, Reduced graphene oxide, Platelet extracellular vesicles (pEVs), Wound healing, Antiinflammation

## Abstract

Impaired wound healing is a significant complication of diabetes. Platelet-derived extracellular vesicles (pEVs), rich in growth factors and cytokines, show promise as a powerful biotherapy to modulate cellular proliferation, angiogenesis, immunomodulation, and inflammation. For practical home-based wound therapy, however, pEVs should be incorporated into wound bandages with careful attention to delivery strategies. In this work, a gelatin-alginate hydrogel (GelAlg) loaded with reduced graphene oxide (rGO) was fabricated, and its potential as a diabetic wound dressing was investigated. The GelAlg@rGO-pEV gel exhibited excellent mechanical stability and biocompatibility in vitro, with promising macrophage polarization and reactive oxygen species (ROS)-scavenging capability. In vitro cell migration experiments were complemented by in vivo investigations using a streptozotocin-induced diabetic rat wound model. When exposed to near-infrared light at 2 W cm^− 2^, the GelAlg@rGO-pEV hydrogel effectively decreased the expression of inflammatory biomarkers, regulated immune response, promoted angiogenesis, and enhanced diabetic wound healing. Interestingly, the GelAlg@rGO-pEV hydrogel also increased the expression of heat shock proteins involved in cellular protective pathways. These findings suggest that the engineered GelAlg@rGO-pEV hydrogel has the potential to serve as a wound dressing that can modulate immune responses, inflammation, angiogenesis, and follicle regeneration in diabetic wounds, potentially leading to accelerated healing of chronic wounds.

**Supplementary Information:**

The online version contains supplementary material available at 10.1186/s12951-023-02068-x.

## Introduction

It is well established that chronic exposure to a glucose-rich environment induces various physiological and pathophysiological changes. Prolonged hyperglycemic conditions can lead to severe diabetic complications, damaging the pancreatic β-cell, inducing insulin resistance, and causing numerous complications, one of which includes chronic diabetic wounds [1]. Chronic diabetic wounds are characterized by persistent inflammation and diminished production of the extracellular matrix (ECM) [2, 3]. Restrained vascularization in such wounds, attributed to restricted blood flow, hampers the migration of leukocytes, keratinocytes, fibroblasts, and endothelial progenitor cells to the wound site, thus slowing down wound closure. Another distinguishing hallmark of chronic diabetic wounds is the presence of biofilm*-*forming bacteria, which are considered a significant impediment to the healing of diabetic wounds [4].

Recently, the use of hydrogels or functional biomaterials to promote wound healing has gained increased attention, with promising results having been reported by several teams. Wang et al. developed an integrated microfluidic device capable of simultaneously detecting multiple antibiotics [5]. In another investigation, Xu et al. created a polyphenol-modified chitosan hybrid hydrogel with antimicrobial and antioxidant properties, designed to accelerate the healing process of diabetic wounds [6]. Additionally, Liu et al. established the efficacy of a Janus hydrogel possessing both antibacterial and angiogenesis functions, contributing to the enhanced healing of diabetic wounds [7].

Due to their structural similarity to the ECM [8–11], polymeric hydrogels based on gelatin (Gel) and alginate (Alg) are widely used for wound protection [12, 13]. Zhao et al. proposed a gelatin acrylamide as support to keratinocyte growth, differentiation, and stratification, which resulted in the development of reconstructed multilayered epidermis [14]. This gelatin acrylamide hydrogel also exhibited excellent cell viability, increased cell adhesion and proliferation. Similarly, Nicholas et al. reported encouraging findings in skin regeneration using a pullulan-gelatin hydrogel, observing enhanced angiogenesis and positive effects on skin healing and tissue regeneration [15]. However, one of the primary limitations of most hydrogels is their poor mechanical properties over time. Although using higher polymeric concentrations can enhance the fragile nature of hydrogels, this often compromises the porosity and desired cell-friendly morphology [16, 17]. As a result, these hydrogels have limited therapeutic efficacy, triggering substantial recent efforts towards alternative strategies for drug loading into these hydrogels [16, 18, 19].

**Scheme 1:**
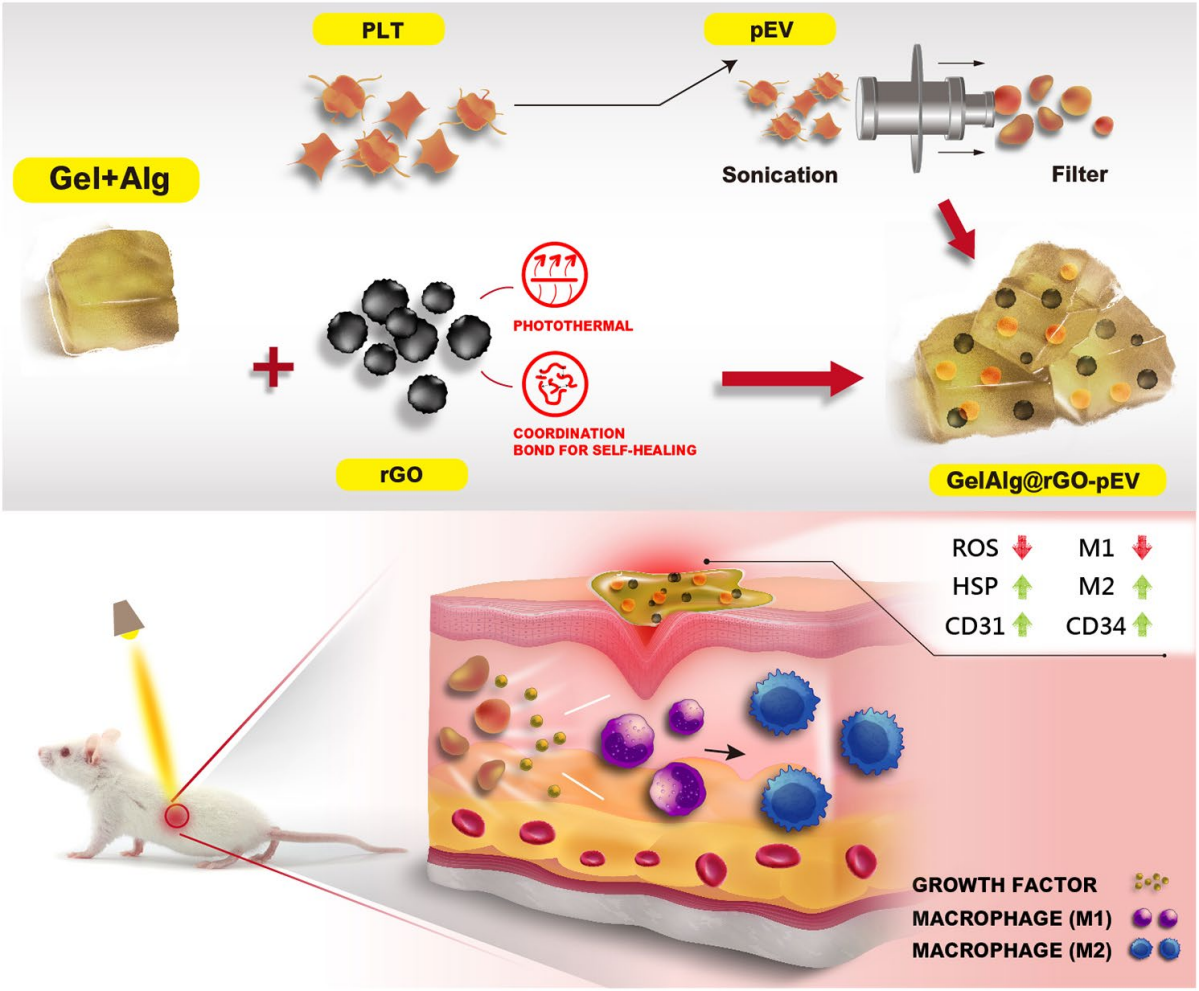
Schematics of the formation of a multicomponent gelatin-alginate (GelAlg) based hydrogel loaded with human blood platelet-derived EVs (pEVs) and reduced graphene oxide (rGO) for accelerated diabetic wound healing

Biocompatible Gel has in parallel found extensive utility in hydrogel production owing to its distinctive ability to generate transparent gels and its high-water absorption ability [20]. Its degradation at temperatures generally > 37 °C results in localized release of encapsulated therapeutics [21]. The stability of Gel at higher temperatures is, however, not provided and different hydrogel formulations need to be considered for temperature-based drug release. Sodium alginate (Alg) shows a decomposition temperature > 100 °C [22]. Given its abundant occurrence, cost-effectiveness, and straightforward processing methods, extensive research has been conducted on Alg for the creation of hydrogel matrices [23] and will be considered here for the formation of a temperature-stable GelAlg hydrogel The developed GelAlg hydrogel was further loaded with reduced graphene oxide (rGO) to provide a hydrogel with photothermal properties (Scheme 1). The incorporation of rGO enhances the mechanical modulus of the hydrogel through polymeric coordination bonding and adjusts the micromorphological structure of the gel [13, 24]. The strong light absorption ability of rGO in the near-infrared (NIR) region also makes it an excellent photothermal agent [25–32], with the possibility for photothermal-derived hyperthermia to form heat shock proteins (HSP) [33]. Additionally, human platelet-derived extracellular vesicles (pEVs) should be advantageously integrated into the gels due to their reported positive biological effects in diabetic wound healing [34, 35] studies. Extracellular vesicles (EVs) are 50–800 nm lipid-bilayer membrane-coated particles released by cells into their surrounding environment. They have demonstrated their ability to modulate cell proliferation, immunity, angiogenesis, and inflammation [36, 37]. Specifically, pEVs, which carry a diverse array of platelet growth factors, including a substantial amount of pro-angiogenic vascular endothelial growth factor (VEGF) [38], can be readily prepared from clinical-grade human platelet concentrates and lysates [38–40]. This makes pEVs particularly suitable for translational applications, and they are now attracting considerable interest as a biotherapy in various clinical fields [39]. Chronic wounds often exhibit decreased levels of VEGF, which suggests that the use of pEVs, which are loaded with VEGF and other trophic factors [38, 39, 41], could serve as an efficient, cost-effective alternative to single recombinant growth factors for wound healing modulation [42]. Topical applications of these recombinant growth factors have demonstrated limited effectiveness in clinical settings largely due to their low in vivo stability and restricted absorption through the skin surrounding the wound [43]. pEVs, together with EVs derived from megakaryocytes, their precursors in the bone marrow, are the most abundant EVs in human blood [44–46] and offer promising potential for wound healing, as recently reported [47, 48]. The realistic translational potential of p-EVs is well illustrated by the recent publication of a double-blind placebo-controlled trial of the safety and potential efficacy of pEVs for wound healing [41]. Indeed, the incorporation of these pEVs into hydrogels, as evaluated in this work, provides an advantage for in vivo applications thanks to the enhanced wound healing effect and protection of the multiple platelet trophic factors they contain against enzymatic degradations.

## Materials and methods

### Materials

Gelatin (Gel) from bovine skin (type B) (CAS#: 9000-70-8) and sodium alginate (Alg) (CAS#: 9005-38-3) were purchased from Sigma-Aldrich (St. Louis, MO, USA). Reduced graphene oxide (rGO) was obtained from Bioman Taiwan (New Taipei City, Taiwan)). All other biochemical reagents were acquired from Thermo Fisher Scientific (Cleveland, OH, USA) and were of high-performance liquid chromatography (HPLC) grade.

### Synthesis and characterization of GelAlg@rGO-pEV

Gelatin (30 g) was dissolved at 60 °C in PBS (300 mL, pH 7.4). Alginate (20 g) was dissolved in water (750 mL) at 40 °C. To obtain pure Gel and Alg, they were dialyzed against DI water for three days (MWCO: 3.5 kDa), centrifuged for 5 min at 8.000 rpm, filtered (0.22-micrometer pore size), and lyophilized at − 80 °C. The lyophilized Gel and Alg powders were stored at − 20 °C for subsequent use. A mixture was prepared by evenly combining 10% Gel and 200 µg mL^− 1^ rGO. Subsequently, 7.5% Alg and the prepared pEVs (2 mg ml^− 1^) were added to the mixture and stirred at 40 °C for 30 min.

### Characterization

#### Scanning Electron Microscopy (SEM)

images were obtained with an FE-SEM-Nova Nano 450 (Nova Nano, USA) equipped with energy dispersive spectrometry (EDS). **Sized and surface potential** measurements were performed with a Zetasizer (Malvern Zetasizer Nano-ZS90 instrument, Malvern, UK, 22 °C). **Fluorescence spectrometric** measurements were conducted on Leica Biosystems (Germany).

#### Photothermal properties

Photothermal properties were determined in 96-well plates at different rGO concentrations or hydrogel samples (GelAlg or GelAlg@rGO) under 5 min NIR irradiation at 808 nm at 2.0 W cm^− 2^ (Optoelectronics Tech Psu-iii, China). Distilled water without rGO was used as a negative control. The temperature and thermal images of the solutions were determined and collected during irradiation with an infrared (IR) thermal camera (MET-FLTG300 + 2 NIR, S.E.A.T, Taiwan).

#### Mechanical characterization

A dynamic texture analysis (TA. XT Plus, UK) was employed to evaluate the compression modulus of the hydrogels at room temperature. For GelAlg and GelAlg@rGO, the samples with circles of 10 mm in diameter were used. Data were displayed as compressive stress vs. working time or distance. A rheometer (AR2000ex, TA Instruments, stress control) was employed to measure the hydrogels’ rheological properties ranging from 0 to 40 °C.

**Swelling and degradation behavior of hydrogels**: The swelling and degradation behavior of the hydrogels were assessed at 37 °C in a pH 7.4 1 × PBS solution. Hydrogels were soaked in 1 × PBS (pH 7.4) at 37 °C. At designated time points, samples were removed and weighed. Before weighing, excess water was drained off with filter paper. The water swelling ratio of the hydrogel samples was calculated according to Eq. (1): [49].

1 $$Swelling{\rm{ }}rate{\rm{ }}\left( \% \right){\rm{ }} = {\rm{ }}[(Ws{\rm{ }} - {\rm{ }}{W_0})/{W_0}]{\rm{ }} \times {\rm{ }}100{\rm{ }}\left( \% \right)$$

in which Ws is the weight in the swollen state, and W_0_ is the initial weight of the hydrogel sample.

The degradation rate of the hydrogel sample was determined according to Eq. (2).

2 $$Degradation{\rm{ }}rate{\rm{ }}\left( \% \right){\rm{ }} = {\rm{ }}\left[ {\left( {{W_0}-{\rm{ }}Wt} \right)/{W_0}} \right]{\rm{ }} \times {\rm{ }}100{\rm{ }}\left( \% \right)$$

in which Wt is the weight after degradation, and W_0_ is the initial weight of the lyophilized hydrogel sample.

### Drug release evaluation

Bovine serum albumin (BSA) was covalently conjugated to Cy5-N-hydroxysuccinimide (NHS) ester by mixing BSA (4 mg/mL) with Cy5-NHS-ester (0.1 mg/mL) and keeping the reaction under stirring for 2 h at room temperate. Separation of unreacted dye was achieved by dialysis in a 100 kDa dialysis bag for 3 days with sterile DI water, followed by 2–3 times buffer (DI water) change at room temperature.

Gel (100 mg mL^− 1^), Alg, (75 mg mL^− 1^), and rGO (200 µg mL^− 1^) were mixed with Cy5-BSA (2 mg mL^− 1^) at room temperature for 30 min and dialysis 2 days to remove impurities to form GelAlg@rGO-Cy5-BSA.

To assess passive drug release, 200 mg of the formed sample was added to a 3500-Da dialysis bag and dialyzed against pH 7.4, 1 × PBS, with liquid surrounding the dialysis bag being collected.

### Biological assays

#### Preparation of pEVs

Human platelets were obtained from Innovative Research (Novi, MI, USA). They were centrifuged for 10 min at 1500 ×*g* and 22 °C to pelletize the platelets and remove the plasma supernatant. The resultant platelet suspension (2 mg mL^− 1^, pH 7.4 in 1 ×PBS) was then subjected to ultrasonication (at 40% power of sonication bath) for 30 min and filtered through a sterile syringe filter (with a 0.22-µm pore size) to obtain pEVs.

The platelet samples, post sonication and filtration, were processed using standard protocols, separated by sodium dodecyl sulfate-polyacrylamide gel electrophoresis (SDS-PAGE) under non-reducing conditions. Lyophilized samples were dissolved in acetic acid (0.5 M) at 5 mg mL^− 1^. These samples were then heated to 95 °C for 10 min to completely denature the proteins, and 5 µg of each protein sample was loaded into the gel. Additionally, 4 µL of a protein marker (molecular weight ladder ranging from 10 to 245 kDa) was loaded in separate wells. Electrophoresis was performed at 90 V until the front line reached the bottom portion of the gel. After running, the gel was stained with a Coomassie (10% acetic acid, 0.125% Coomassie blue R 250, and 50% methanol) staining solution for 40 min, and then soaked three times in a destaining solution (7% acetic acid and 5% methanol) for 0.5 h each and once with DI water overnight.

The morphology of purified pEVs was examined using a high-resolution transmission electron microscope (TEM HT-7700, Hitachi, Japan). The particle size and concentration of pEVs were measured using state-of-the-art NanoSight NS300 detectors (NTA NanoSight NS300, Malvern Panalytical, UK). Furthermore, the zeta potential and particle size of pEVs were characterized using a highly precise Malvern Laser Particle Size Analyzer (DLS ZS90, Malvern Panalytical, UK).

#### Cell cytotoxicity

The cytotoxicity of different samples (pEVs, GelAlg@rGO and GelAlg@rGO-pEV) was evaluated by the MTT assay using National Collection of Type Cultures (NCTC) clone 929 from connective mouse tissue (L929) for 24 h in full Dulbecco’s modified Eagle medium (DMEM) (containing 10% FBS, at 37 °C). L929 cells were grown at 37 °C and 5% CO_2_ in DMEM and were seeded in 96-well plates at an initial density of 10^4^ cells/well. Once L929 cells had adhered to the plate, various concentrations (20 µg mL^− 1^, 200 µg mL^− 1^, and 2 mg mL^− 1^) of the biomaterial sample extract were added to the above-mentioned well plates, and healthy plates without a sample extract were used as a control. The limit for cytotoxicity is 70% viability of control cells, as per ISO 10,993. In the MTT test, samples were incubated with an aqueous solution of 250 µL of MTT together with 750 µL of complete medium at 37 °C for 4 h, in a 5% CO_2_ atmosphere; formazan crystals were dissolved in dimethyl sulfoxide (DMSO). The absorbance of the reacted solutions was determined at 570 nm on a microplate reader. Cell viability was also characterized with a live/dead kit. First, L929 cells were incubated with extracted samples and washed for 10 min with PBS. Then, a staining reagent was prepared with 5 µL of a 4 mM calcein-AM stock solution plus 20 µL of a 2 mM ethidium homodimer (EthD-1) stock solution in 10 ml of PBS. Treated L929 cells were immersed in staining reagents in the dark for 45 min. After washing with a PBS solution twice, cell morphological structures were imaged with a fluorescence microscope (Leica, Germany). Fluorescence images of live/dead cells were determined by green colour indicating living cells and red colour indicating dead cells.

#### Macrophage polarization

Fluorescence microscopy (Leica, Germany) was used to evaluate macrophage polarization in RAW264.7, a macrophage-like, Abelson leukemia virus-transformed cell line derived from BALB/c mice) obtained from American Type Culture Collection (ATCC, Manassas, VA, USA). The cells were treated with 100 ng/mL lipopolysaccharide (LPS) for 1 day before being cultivated with various samples for 3 days. Treated RAW264.7 cells were washed with a pH 7.4 solution. After being fixed for 30 min with 4% paraformaldehyde, cells were treated at 4 °C for 1 h with a fluorescent-conjugated anti-cluster of differentiation 206 (CD206) (M2) antibody (FITC anti-mouse CD206 (MMR) Antibody, BioLenged, San Diego, CA) or a fluorescent-conjugated anti-CD86 (M1) antibody (APC anti-mouse CD86 Antibody, BioLenged, San Diego, CA).

#### Cellular ROS levels

2,7-Dichlorodihydrofluorescein diacetate (DCFH-DA) was employed to measure cellular ROS levels. L929 and RAW264.7 cells were seeded onto various samples and cultivated for 24 h before being given 100 ng/mL LPS for an additional 24 h. Cells were treated in a 10 µM of DCFH-DA solution at 37 °C for 20 min and were measured by fluorescence microscopy wavelength 490–530 nm.

#### In vitro wound-healing assays

Wound-healing assays were conducted on L929 cells. Culture-Insert 2 wells in a 35-mm µ-Dish (ibidi, Germany) were exploited to assess the in vitro cell migration rate. L929 cells (70 µL) in the medium were seeded at 3 × 10^5^ cells/mL per chamber and cultured for 24 h. Culture inserts were then carefully lifted out, and unattached L929 cells were removed by rinsing the gel twice with warm pH 7.4 PBS. Different samples were then supplemented into each well, and images for in vitro wound healing were immediately acquired. Cells were then incubated for 12 h at 37 °C, and the same fields were reimaged. ImageJ software was utilized to quantify cell migration.

### In vivo evaluations of the wound healing matrixes using diabetic rats

The Animal Ethics and Use Committee of Taipei Medical University approved all animal treatment procedures. Wistar rats (200–240 g) were obtained from Bio-LASCO (Taipei, Taiwan) and were fed and watered for 1 week for acclimatization. As per our previous method, chronic full-thickness dorsal skin wounds were established in diabetic rats [50]. In brief, streptozotocin (STZ, 40 mg kg^− 1^ body weight (BW)/day) was intraperitoneally (i.p.) injected in all Wistar rats for 5 days. A blood glucose meter determines Fasting blood glucose levels (Roche, Taiwan). Rats with fasting blood glucose levels of more than 250 mg dL^− 1^ were chosen to establish full-thickness wounds.

After diabetic rats had exhibited hyperglycemia for 1 week, about 8-mm-diameter full-thickness skin defects were incised on the rat backs using a sterile biopsy punch under anesthesia by inhalant that administered 3% isoflurane. PBS, pEVs, GelAlg@rGO, GelAlg-pEV, and GelAlg@rGO-pEV were topically applied to the created wounds. The wounds were irradiated at NIR (808 nm, 5 min, 2.0 W cm^− 2^) every 2 days for 5 min. Treated wound sites were photographed with a digital camera on days 0, 2, 8, and 14 post-wounding. The remaining wound area (%) was calculated by (the wound area at different time points/wound area on day 0) × 100%. On days 7 and 14 post-wounding, diabetic rats were sacrificed, and the wounded organs and major tissues (heart, liver, spleen, lungs, and kidneys) were harvested and fixed in a 4% paraformaldehyde solution. Fixed tissues were embedded in paraffin for routine histological processing, and sections of about 5 μm of thickness were made. Afterward, histological sections were stained with hematoxylin and eosin (H&E) dye for microscopic observation. For immunofluorescence (IF) observations, skin sections were blocked with 10% BSA and 0.2% of Triton X-100 for 1 h. Sections were incubated with DCFH-DA and fluorescence-labeled primary antibodies, including an anti-CD86 antibody (1:150), anti-CD206 antibody (1:200), anti-HSP antibody (1:250), anti-CD31 antibody (1:150), anti-CD 31 antibodies (1:150) and anti-CD 34 antibodies (1:150) overnight at 4 °C. After washing three times with PBS, stained sections were incubated with 4′,6-diamidino-2-phenylindole (DAPI) for 3 min at 25 ℃. IF data were assessed using a fluorescence microscope and investigated using ImageJ software.

### Statistical analysis

All experiments were independently repeated at least three times, and quantitative data are presented as the average ± standard deviation (SD). Statistical analysis was performed using GraphPad Prism (GraphPad Software, La Jolla, CA, USA) and analyzed using Student’s *t*-test. A value of *p* < 0.05 (*) was considered statistically significant.

## Results and discussion

### Development and characterization of GelAlg@rGO

**Fig. 1:**
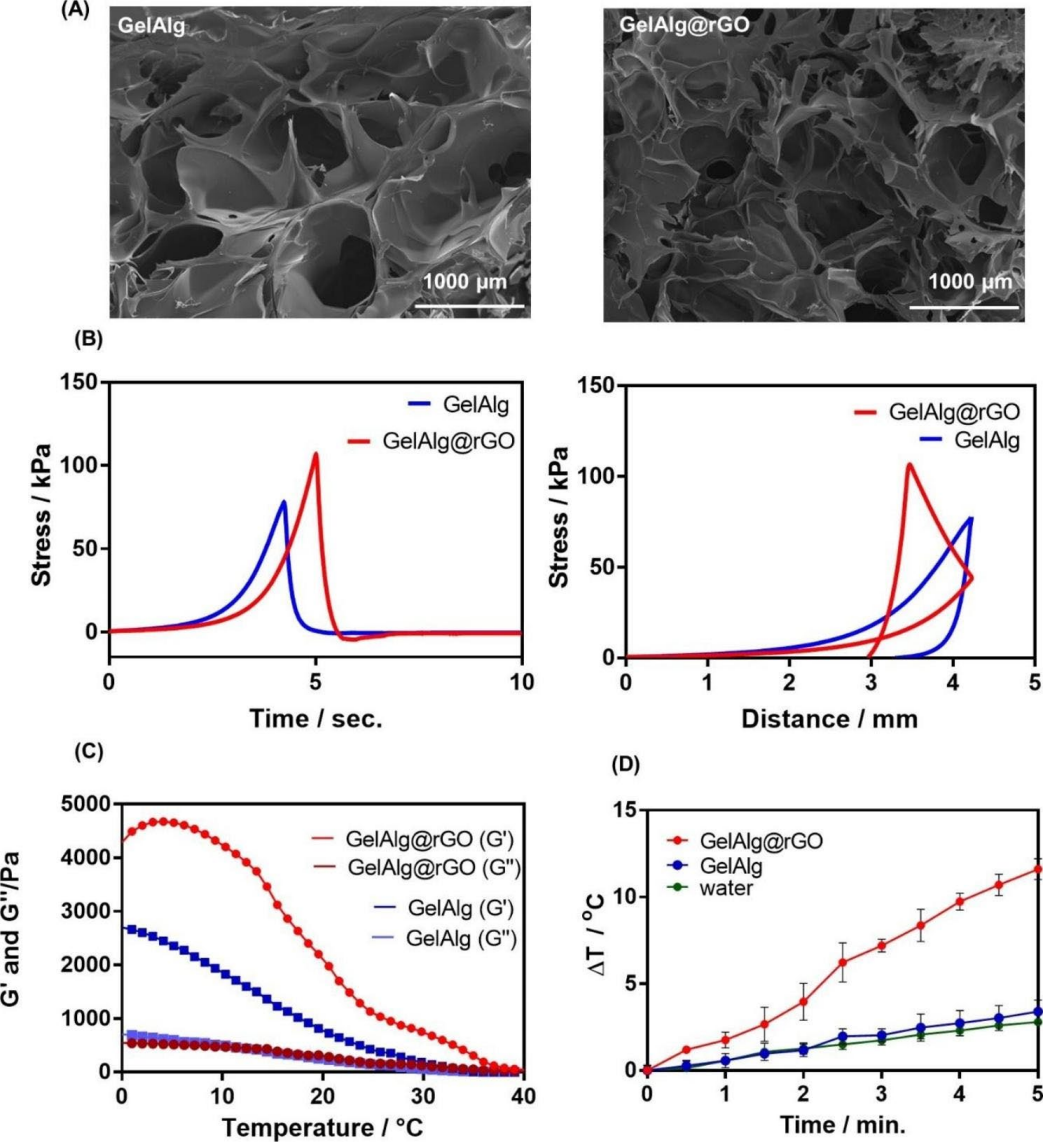
Physicochemical Characteristics of GelAlg and GelAlg@rGO hydrogels. **(A)** SEM images of GelAlg formed from 100 mg ml^− 1^ Gel and 75 mg ml^− 1^ Alg (left) as well as GelAlg@rGO formed from 100 mg ml^− 1^ Gel, 75 mg ml^− 1^Alg and 0.2 mg ml^− 1^rGO (right). **(B)** Texture analysis with time and distance via uniaxial compression experiments. **(C)** Rheological analysis of the hydrogels together with mechanical stability at different temperatures. **(D)** Photothermal analysis of hydrogels when immersed in water for 5 min and illuminated at 808 nm with P = 2 W cm^− 2^

Highly porous bio-scaffold structures that mimic tissue structures and promote nutrient and gas exchange are required for cell proliferation [51]. GelAlg@rGO hydrogels were created by mixing gelatin (Gel) with alginate (Alg) in a 4:3 ratio and leaving the mixture for 30 min at RT. To the formed GelAlg was added 200 µg mL^− 1^ rGO, as this concentration resulted in a temperature change of about 10 °C (Figure S1) when illuminated for 5 min at 808 nm at a power of 2 W cm^− 2^ with no cell toxicity toward L929 cells. The SEM images (Fig. 1A) of GelAlg and GelAlg@rGO hydrogels show highly interconnected pores with porosity of around 61% compared to about 79% for GelAlg.

The mechanical characteristics of GelAlg and GelAlg@rGO were assessed using uniaxial compression experiments and Brookfiel texture analyzer (Fig. 1B**)**. While both hydrogels display linear elastic behavior followed by plastic deformation at higher strain levels (ca. 35%), as demonstrated by the stress-time and stress-distance profiles in Fig. 1B the GelAlg sample exhibits a lower elastic region (78 kPa) compared to 106 kPa in the case of GelAlg@rGO. Achieving high values of moduli is crucial in the context of wound healing applications. Compressive moduli in the range of 100 kPa are beneficial for the growth of keratinocytes or skin cells [52, 53].

The rheological behaviors GelAlg and the GelAlg@rGO were further estimated by determining the energy storage modulus (G’) and loss modulus (G’’) at temperatures ranging from 0 to 40 °C.

Figure 1 C shows that both hydrogels have stable rheological behavior and good mechanical stability at room temperature, with their G’ and G” remaining in equilibrium from 0 to 22 °C with the storage moduli (G’) of both samples being more remarkable than their loss moduli (G”). The GelAlg hydrogel exhibits a decrease from ≈ 2700 Pa to 0 Pa when reaching the skin temperature of about 33 °C [54], suggesting insufficient mechanical strength on the skin. Conversely, GelAlg@rGO gels display a decrease in G’ from ≈ 4500 Pa to 500 Pa when reaching the skin temperature °C and are much better adapted for skin adhesion. These findings demonstrated that the presence of rGO in this system was positively correlated with the elastic modulus of the hydrogel, resulting in increased mechanical strength. Previous studies have documented the mechanism and behavior of GO-intercalated energetic coordination polymers with improved thermostability [55]. The photothermal properties of different GelAlg@rGO hydrogels when illumination for 5 min with a NIR at 808 nm at a power of 2 W cm^− 2^ are shown in Fig. 1D and shows that the presence of rGO in the GelAlg hydrogel integrates photothermal properties.

**Fig. 2:**
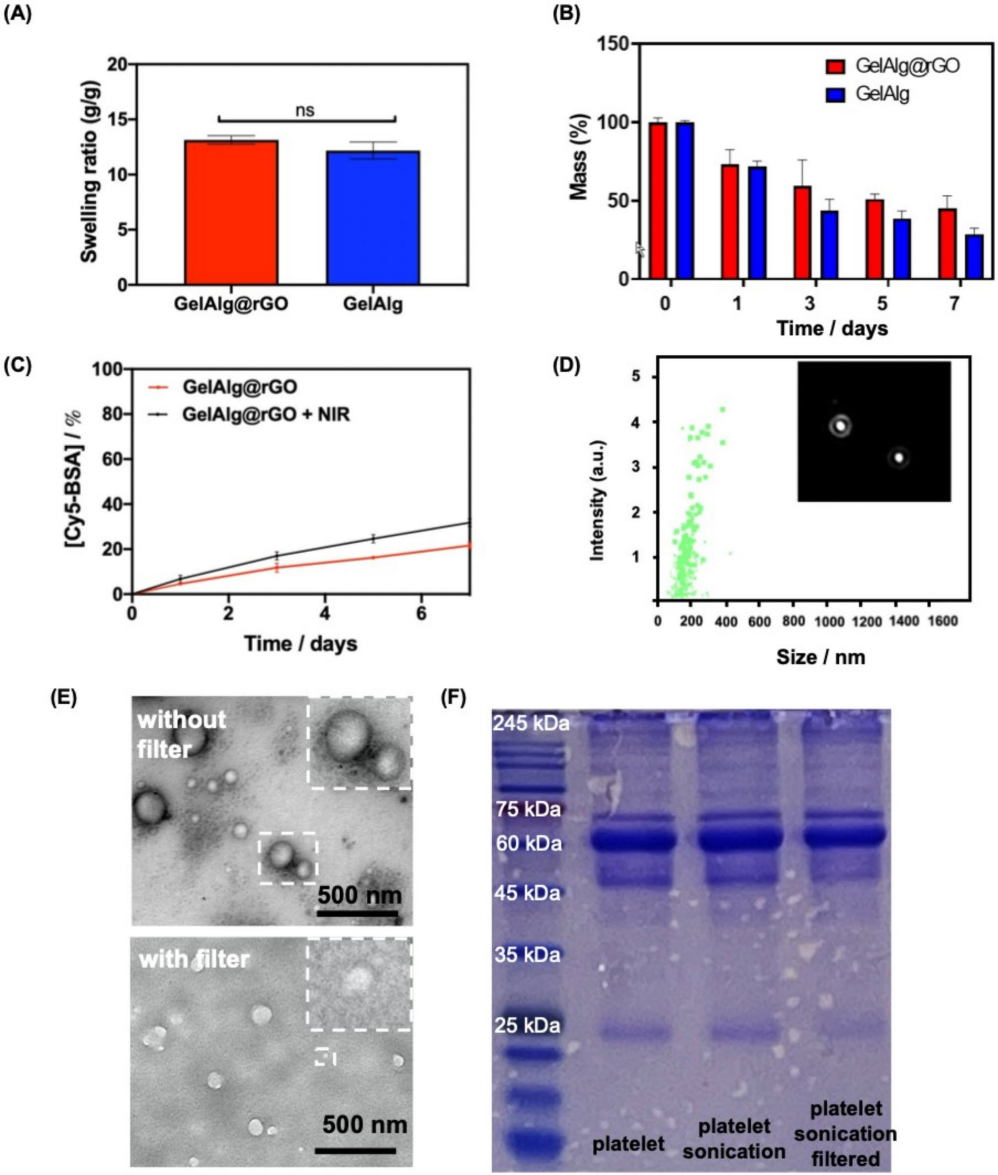
Swelling and drug release properties of GelAlg and GelAlg@rGO hydrogels. **(A)** Change of mass ratio upon immersion in water for 24 h. **(B)** Mass loss in percentage of hydrogels as a function of time. **(C)** Release of Cy5-BSA loaded GelAlg@rGO hydrogels with and without NIR activation (808 nm, 2 W/cm^2^). **(D)** The nanoparticle tracking analysis (NTA) of filtrated-prepared pEVs. **(E)** TEM images of ultrasonicated platelets without or with filtration. **(F)** SDS-PAGE analysis of protein samples (platelets, ultrasonicated platelets, and ultrasonicated platelets with filtration)

To ensure the formed hydrogel’s utility to hold substantial amounts of water and make it suitable for skin wound dressings, the swelling properties were assessed in 1 × PBS at 37 °C (Fig. 2A). Both hydrogels reached swelling equilibrium after 24 h, with a swelling ratio of at least 10 g/g with the presence of rGO having no significant effect on the water uptake. Both hydrogels degraded however rapidly when immersed in 1 × PBS at 37 °C over the first three days, followed by a slower degradation thereafter (Fig. 2B). On day 7, the GelAlg hydrogel showed a remaining weight of approximately 29%, while in the case of GelAlg@rGO, 45% was observed.

### Loading and release of a model drug such as BSA from GelAlg@rGO

First, Cy5-BSA was utilized as a model molecule for the release capacity of pEVs from GelAlg@rGO hydrogels. Immersion of Cy5-BSA loaded GelAlg@rGO hydrogels shows that after 7 days only about 22% of Cy5-BSA was released corresponding to a flux of about. 4.23 µg h^− 1^ cm^− 1^) (Fig. 2C). In the case of light activation (808 nm, 5 min), an increase to 32% was released, due to hydrogel swelling and drug release, remaining rather low. Based on previously published literature, BSA molecules are carrying, like p-EVs, negative charges at physiological pH 7.4 [56]. It was speculated that BSA with a low pI might effectively bind to a Gel-based hydrogel matrix by electrostatic attraction [57]. As per previously reported findings, the release rate of BSA decreased, which is due to the strong ion electrostatic attraction between BSA and polymeric matrix [58] or composite matrix [59]. BSA-laden hydrogel system thus possibly exhibits a slow-release effect.

### Loading of with pEVs

pEVs loaded gels were investigated as follows. According to the NTA data, the concentration of the purified and filtered pEVs was approximately 1.03 × 10^11^ particles mL^− 1^ (Fig. 2D). TEM images revealed that the unfiltered pEVs suspension had a non-uniform particle distribution (Fig. 2E**)**, while after filtration (0.22 μm, polyvinylidene difluoride) a homogeneous pEVs distribution was obtained. Independent of the method used for the generation, the pEVs were negatively charged with a zeta potential of − 14 ± 2 mV. These pEVs were subsequently loaded into GelAlg and GelAlg@rGO. The total protein profile was analyzed by SDS-PAGE (Fig. 2F), confirming the presence of proteins with a molecular mass of 66.5 kDa, likely corresponding to albumin [60].

### Cell toxicity of GelAlg@rGO-pEV to L929 cells

**Fig. 3:**
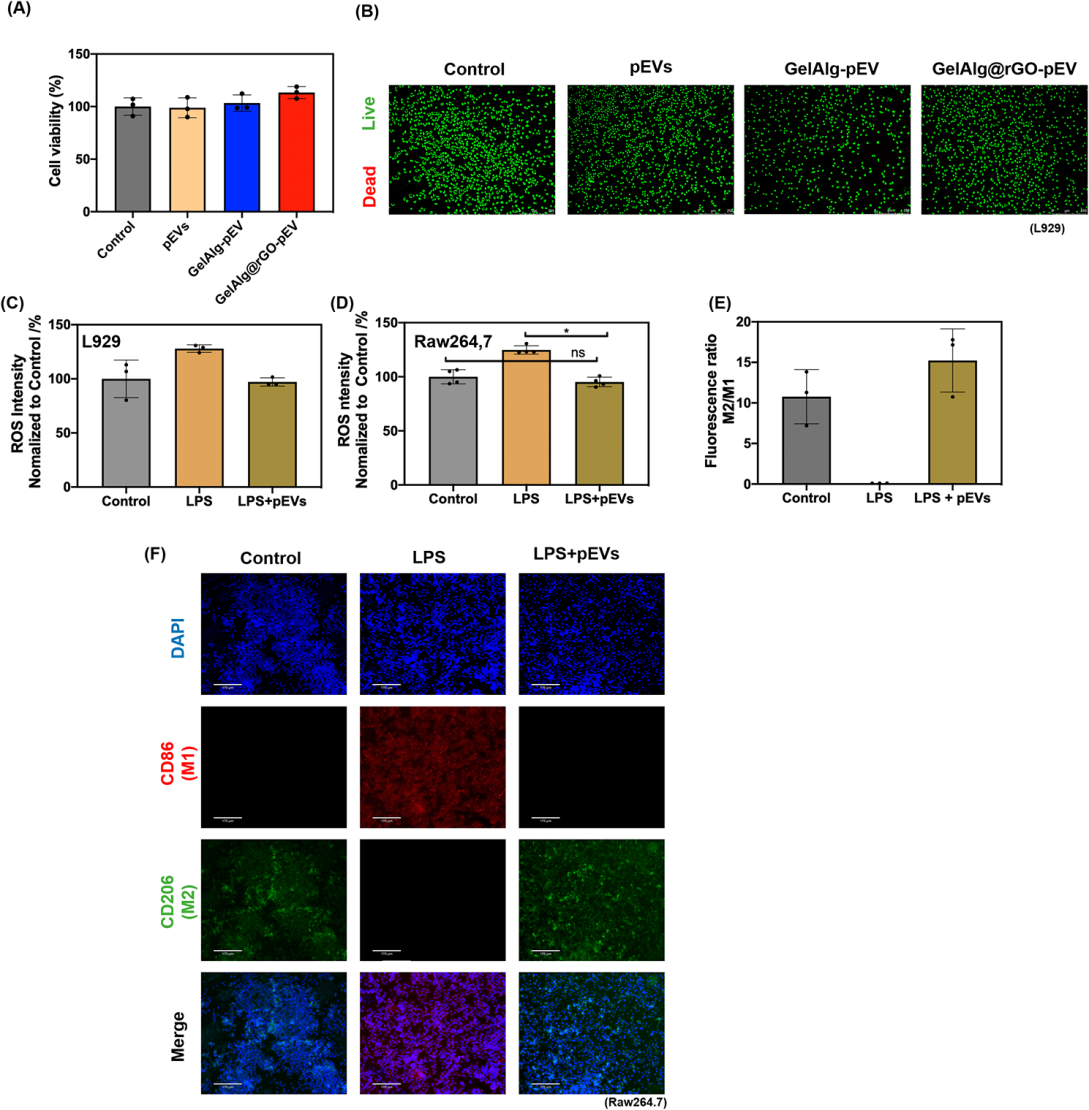
Cell viability, ROS scavenging properties, and immune-cell modulating responses. **(A)** Cell viability of pEVs (2 mg mL^− 1^), and pEVs loaded GelAlg and GelAlg@rGO determined using the MTT (3-[4,5-dimethylthiazol-2-yl]-2,5 diphenyl tetrazolium bromide) assay based on the conversion of MTT into formazan crystals by living cells after 24 h and **(B)** live/dead assays of PBS, pEVs and pEVs-loaded hydrogels with 5 min of NIR irradiation. **(C)** Intracellular ROS fluorescence intensities of L929 and **(D)** Intracellular ROS fluorescence intensities on RAW264.7 cells when in contact with LPS and LPS + pEVs using DCFH. **(E)** The ratio of the fluorescence signal of CD206 (M2 macrophage biomarker) over CD86 (M1 macrophage biomarker). Data are represented as mean +/- SD, with n = 3 replicates; (ns > 0.05. *p < 0.05). **(F)** The cells were treated with 100 ng/mL lipopolysaccharide (LPS) for 1 day before being cultivated with various samples for 3 days. Fluorescence microscopic detection of the macrophage polarization

The cytotoxicity of the hydrogels was evaluated on monolayer cultures of L929 cells, selected as skin connective tissues. The viability of L929 cells was > 80% after incubation for 24 h with pEVs (2 mg mL^− 1^), pEVs loaded, and GelAlg@rGO samples with 5 min of NIR irradiation, suggesting no apparent cytotoxicity **(**Fig. 3A**)**.

Indeed, the group treated with GelAlg@rGO-pEV demonstrated a slightly increased cell proliferation compared to the control group (Fig. 3A), which comprised only the cell medium and cells. This observed enhancement in cell proliferation could be attributed not solely to the presence of pEVs but also to the synergistic biological effects of rGO. The combined influence of pEVs and rGO contributed to the observed beneficial effect on cell proliferation, emphasizing the significance of their cooperative action in promoting cellular growth and activity. A recent study has substantiated the pivotal role of GO as a crucial component in tissue engineering scaffolds, primarily attributed to its unique ability to modulate cellular behavior [61]. This research unveils the enhanced biocompatibility, cell proliferation, and targeted cellular differentiation facilitated by the synergistic effect between GO and polypeptides or proteins. In line with previously published findings, this paper showcases the successful fabrication of a bioscaffold composite, exhibiting remarkable properties for tissue regeneration, including injectable stem cell composite hydrogel composed of silk fibroin (SF) and graphene oxide, as investigated by Wang et al. [62]. Notably, this composite also fosters sustained growth, proliferation, and differentiation of stem cells. These findings underscore the immense potential of protein-GO composites as a cutting-edge approach for advanced bioengineered tissue applications.

The quantitative live/dead assay further supported the results of the MTT analysis (Fig. 3B), indicating that the plasma membrane integrity and cellular activities were preserved. Indeed, the proportions of green live cells in each experimental group were quite close to that of the control group, which proved the excellent biocompatibility and negligible cytotoxicity towards skin cells.

Based on previously published findings on Gel hydrogels, cell proliferation assessed with the MTS assay over a span of 7 days shows that cell viability is favorable for Gel hydrogels at a concentration of 150 mg ml^− 1^ gelatin [63]. In the case of Alg based gels, cell viability was found to exceed 90%, in the case of a 3.75%/7.5% Alg/Gel composition. This composition emerges as highly suitable for optimal cell growth [64].

Cell viability was examined in this work through an MTT assay involving the cultivation of osteoblasts on substrates containing 1.5 wt% rGO-infused chitosan (CS) for durations of 1, 3, and 5 days. The observed elevation in MTT activity with progressive incubation time provides clear evidence of ongoing proliferation [65]. Building upon prior research, it is evident that a concentration of approximately 10^11^ particles mL^− 1^ of pEVs resulted in heightened viability, accelerated wound healing, intensified proliferation markers, and an enhanced rate of cellular adhesion [38].

In a preceding study, employing a relatively elevated dose (4.8 W/cm^2^ for 15 min) of NIR (830 nm) laser irradiation, a noteworthy increase in cellular ATP was observed within the laser-treated cerebral cortex region compared to the untreated area [66].

### ROS scavenger ability of pEVs to L929 and RAW264.7 cells

Excessive reactive ions species (ROS) production in wound sites impedes the proliferation and survival of skin cells, and ROS-scavenging abilities are an important aspect of wound bandages [67]. To mimic the oxidative stress microenvironment in vitro, L929 (Fig. 3C**)** and RAW264.7 (Fig. 3D**)** cells were treated with liposaccharides (LPS) only or when pEVs (2 mg mL^− 1^) were added and the ROS-scavenging abilities of samples evaluated using 2′,7′-Dichlorofluorescein diacetate (DCFH-DA), a ROS-specific fluorescent prob. In the presence of pEVs, a significantly lower fluorescence intensity than the LPS positive control was observed (*p* < 0.05) in both cases.

### Immune cell-modulating response

Macrophages have a vital function in the immune response and play a key role in all phases of wound healing. As wounds heal, the local macrophage population transitions from predominantly pro-inflammatory (M1-like phenotypes) to anti-inflammatory (M2-like phenotypes) [68]. Accurate and timely conversion of M1-like phenotypes toward anti-inflammatory M2 macrophages is vital to reestablishing healthy homeostasis [69]. To validate if pEVs can achieve immunomodulation, RAW264.7 macrophages were treated with LPS as an inflammatory trigger [70] first, and the immunoregulatory potential of pEVs was assessed by fluorescence measurements via the expression levels of CD86, a marker of M1-like macrophage, as well as of CD206, an M2-macrophages marker.

Figure 3E shows the results of 3 experiments (images of one representative experiment Fig. 3F) indicating clearly that the addition of LPS results in a low M2:M1 fluorescence ratio. In contrast, the addition of pEVs shows an M2/M1 ratio similar to that of the control. These results suggest pEVs mitigate M1 macrophage polarization and promote M2 macrophage polarization in oxidative stress and inflammation. Indeed, these results are in line with the idea that platelet proteins have anti-inflammatory effects and can inhibit the polarization of M1 macrophages by modulating various signaling pathways [71]. Platelets have emerged as a significant focus in the realm of regenerative medicine due to their ability to release substantial quantities of growth factors and cytokines, such as platelet-derived growth factor (PDGF), TGF-β, and VEGF, and other trophic factors [39]. These bioactive molecules present in platelet biomaterials play a pivotal role in tissue healing and regeneration and have been further demonstrated to exert anti-inflammatory and anti-oxidative activities in various cellular and animal models [38, 72, 73]. Recent studies have also unveiled the potential of nanoscale pEVs in influencing M1-type macrophages through the TGF-β pathway, along with the upregulation of IL-10 signaling [74]. The remarkable anti-inflammatory properties of IL-10 serve as a potent negative feedback regulator, effectively inhibiting pro-inflammatory responses and promoting the transition of macrophages to the M2 phenotype, thereby facilitating wound healing and the pro-repair stage [75].

### In vitro wound-healing abilities

**Fig. 4:**
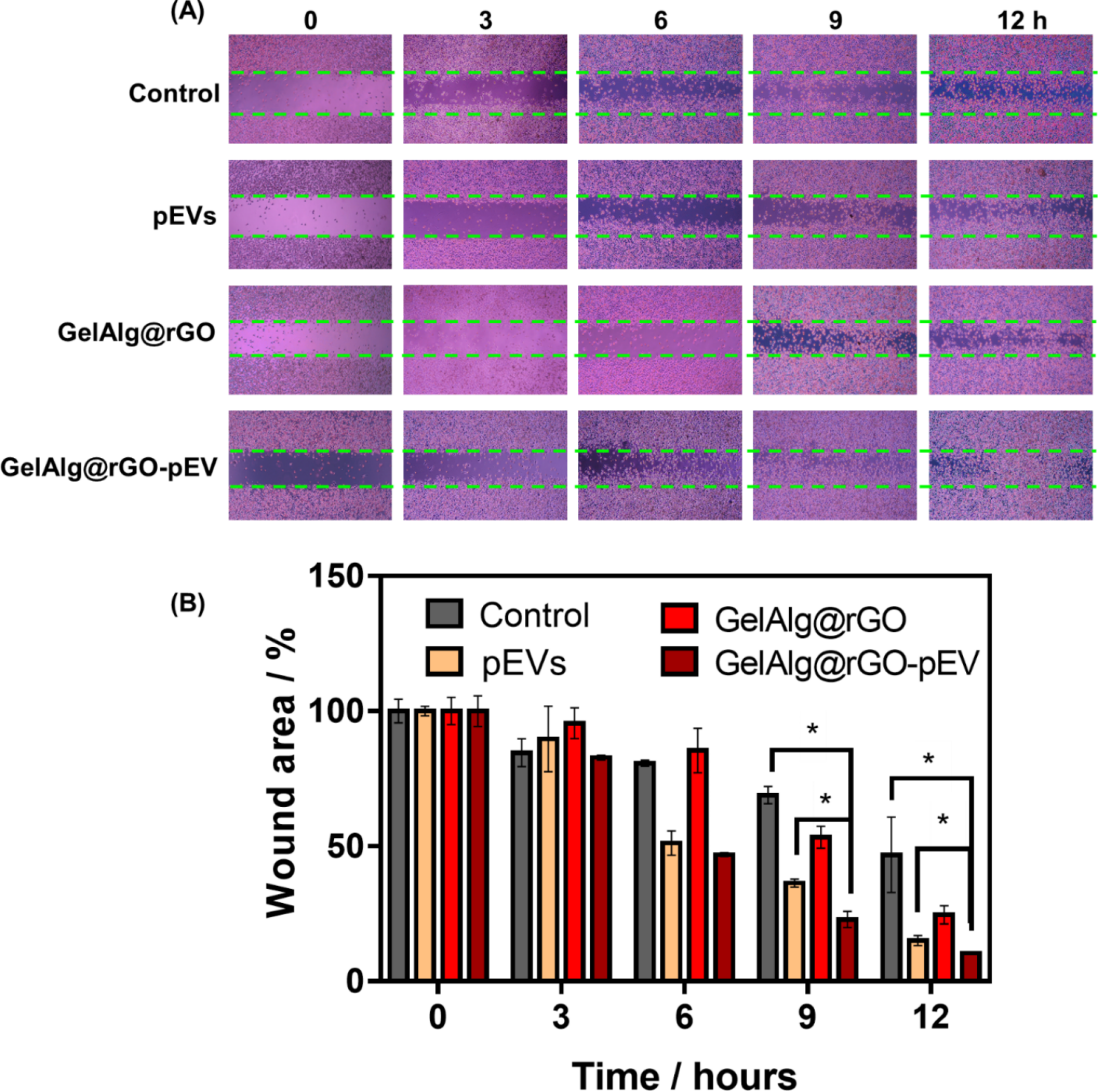
In vitro cell migration consideration on L929 cells using pEVs and GelAlg@rGO-pEV. **(A)** Light microscopic images at L929 cells cultured in PBS (negative control), pEVs (1.03 × 10^11^ particles mL^− 1^), GelAlg@rGO and GelAlg@rGO-pEV with 5 min of NIR irradiation (808 nm, 2 W cm^− 2^) at t = 0. **(B)** Wound closing determined by the relative wound area after 12 h from Fig. 4A. Data are represented as mean +/- SD, with n = 3 replicates; (ns > 0.05. *p < 0.05)

The potential of pEVs-loaded GelAlg@rGO for improved wound healing was examined on L929 cells using a migration assay (Fig. 4A, B). While after 12 h, low migratory levels of L929 cells were observed with pEVs (2 mg mL^− 1^) and the GelAlg@rGO separately, in the case of GelAlg@rGO-pEV considerably more migratory cells were observed, caused by the mildly photothermal hyperthermia, under 5 min of NIR (808 nm, 2 W cm^− 2^) irradiation, compared to other groups including control group (cell plus NIR).

### In vivo evaluations

**Fig. 5:**
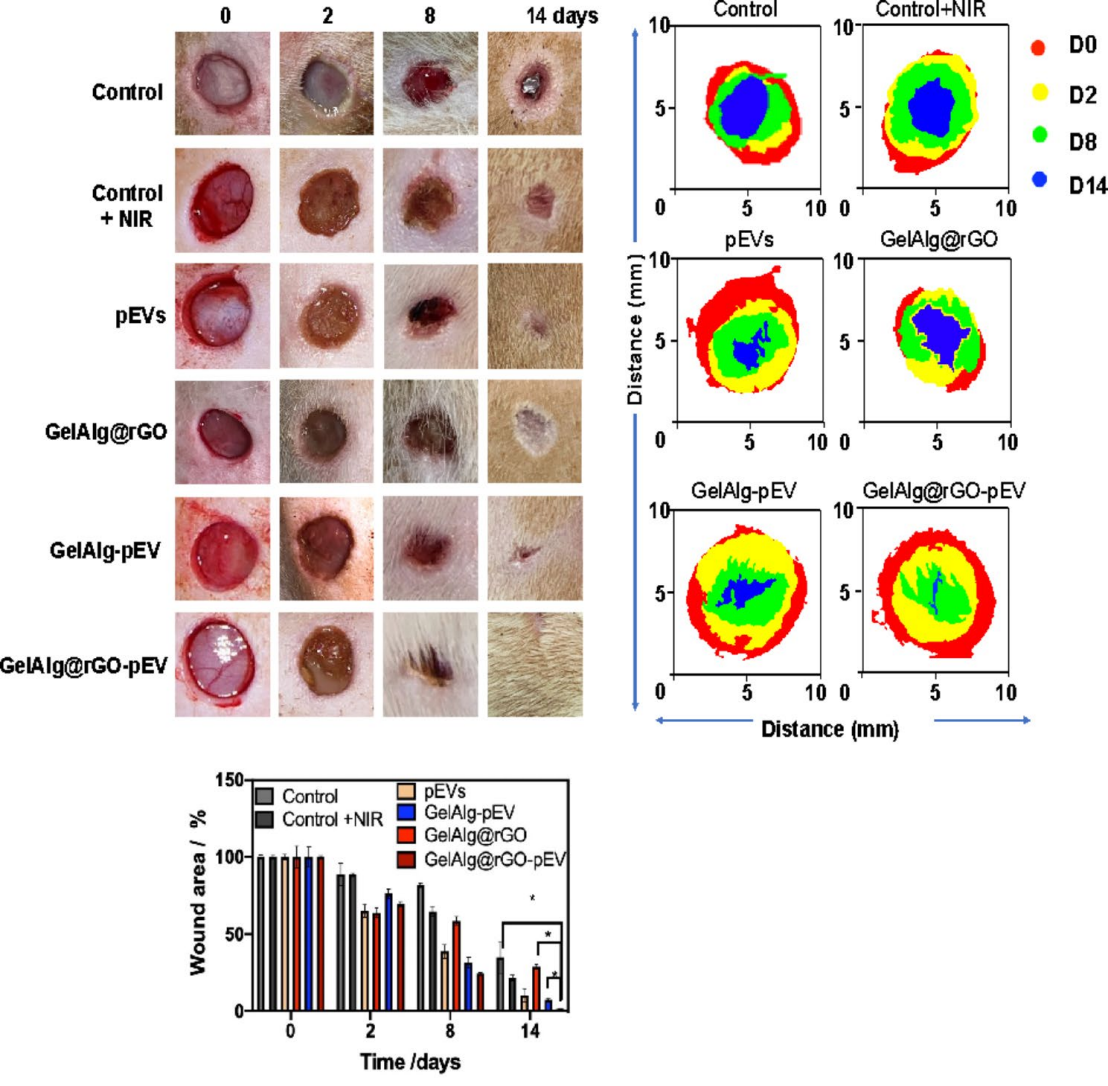
In vivo investigation of wound closes in diabetic rats: Representative gross images of diabetic wounds treated with PBS, pEVs, GelAlg@rGO, GelAlg-pEV, and GelAlg@rGO-pEV with NIR irradiation. The day 0 wound images belonged to the group taken before material administration. The dynamic wound healing areas were calculated using ImageJ software (shown on the right side of the figure). Data are represented as mean +/- SD, with n = 3 replicates; (ns > 0.05, *p < 0.05)

Finally, the possibility of accelerating wound healing in vivo was examined in diabetic rats (Fig. 5). The wound-healing efficiency of pEVs-loaded GelAlg@rGO was more pronounced than those of the PBS, pEVs at the same concentration as GelAlg@rGO, and pEVs-loaded GelAlg. After 14 days of treatment, ca. 35% of the wound area remains unhealed in the PBS negative control group, indicating that the wound cannot close by under this timeframe (Fig. 5). In the case of pEVs-loaded GelAlg@rGO hydrogels, the wound area was reduced entirely after 14 days. These results indicated that the photothermally responsive GelAlg@rGO-pEV hydrogels significantly augmented the healing progression in diabetic rats. These attractive features indicated that the GelAlg@rGO-pEV hydrogels provide preferable diabetic chronic wound-healing efficiency. This is in line with other reports having shown the utility of macrophage-derived exosome-encapsulated microcarrier, in conjunction with mild photothermal therapy [76].

**Fig. 6:**
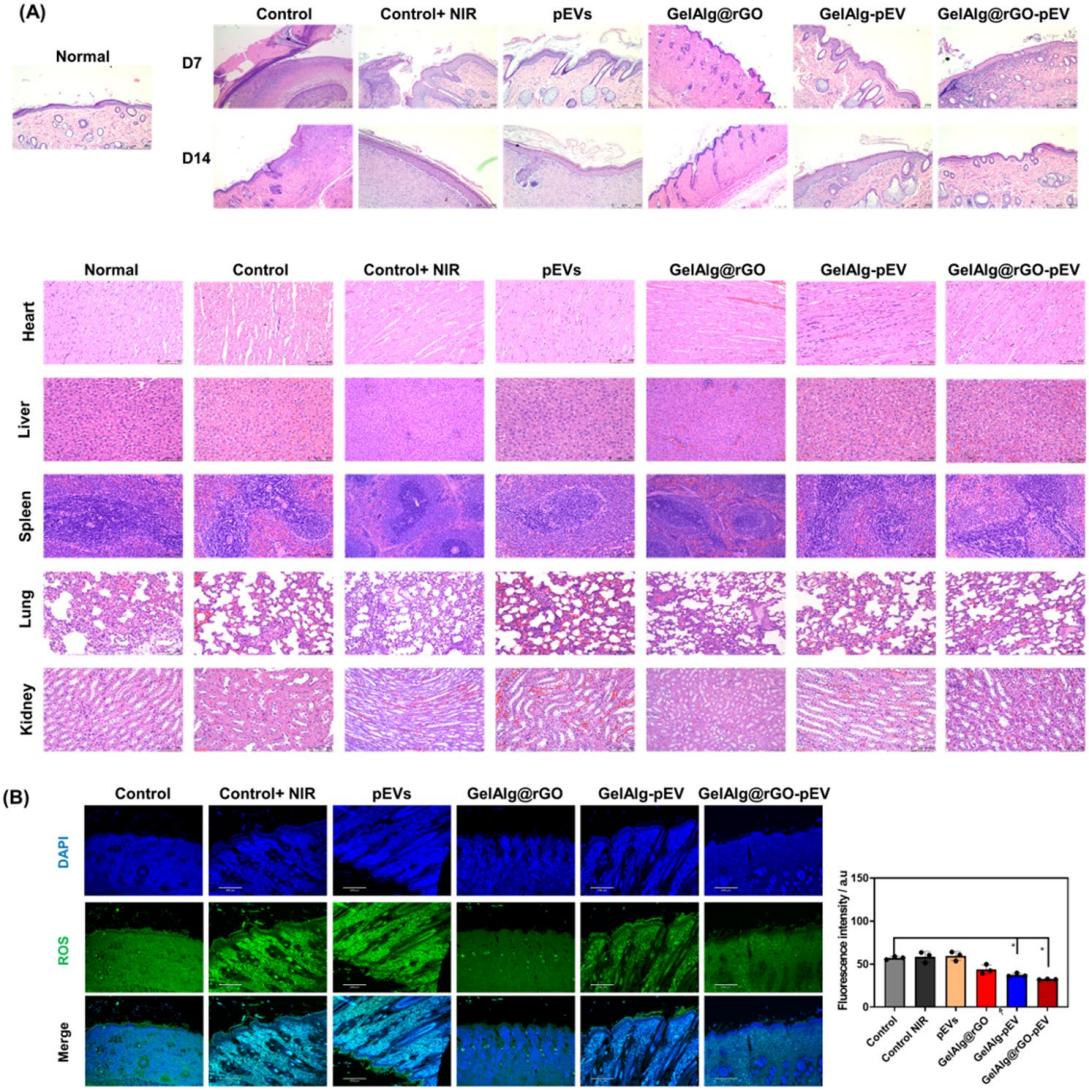
Histological evaluation of the wound after treatment with GelAlg@rGO-pEV; **(A)** H&E staining of diabetic skin wound and organs (heart, liver, spleen, lung, and kidney) after injury and treatments. **(B)** Inflammatory response count in the wounded skin organs by the DCFH-DA assay. Quantification of the fluorescence intensity using ImageJ software. Data are represented as mean +/- SD, with n = 3 replicates, (ns > 0.05, *p < 0.05)

H&E staining was carried out on wounded skin tissues harvested on days 7 and 14 (Fig. 6A). The presence of inflammatory cells, foreign bodies, and necrotic tissue was found in the PBS-negative control group. In the case of pEVs, residual scabs (crusts of exudate and dried blood) were also on day 7. The GelAlg@rGO-pEV group with 5 min NIR irradiation at 2 W cm^− 2^ indicated an improved wound-healing process, which revealed improved organization of the granulation organ and squamous skin epithelium. Non-healing or delayed healing diabetic wounds possess a chronic, excessive inflammatory microenvironment [77]. To further evaluate the in vivo toxicity, major organs, including the heart, liver, spleen, lung, and kidney of diabetic rats, were topically administered with various substances: PBS, pEVs, GelAlg@rGO, GelAlg-pEV, and GelAlg@rGO-pEV, followed by NIR irradiation. After 14 days of administration, the organs were harvested, sectioned, and subjected to histological analysis using H&E staining. Remarkably, no detectable organ damage or signs of inflammatory lesions were observed in the H&E stained sections of the major organs obtained from animals treated with GelAlg@rGO-pEV in conjunction with NIR irradiation (Fig. 6A). These findings suggest that the GelAlg@rGO-pEV formulation did not induce significant toxic side effects in the treated animals.

The extent of inflammation in the wound environment was in addition, assessed using the DCFH-DA assay (Fig. 6B and Figure S2). The PBS group shows a high fluorescence signal due to high ROS concentration due to inflamed wounds. In contrast, the pEVs loaded and NIR activated GelAlg@rGO maintained a low inflammatory microenvironment in the early stages of wound healing, which was encouraging for the rapid transition of the diabetic wound into the proliferation and remodeling stages.

The enhanced therapeutic outcome observed with GelAlg@rGO-pEV can be attributed to the NIR-photothermal properties of rGO. Additionally, the sustained release behavior of the loaded pEVs ensures a continuous and controlled release of therapeutic agents over an extended period [78]. This sustained release allows for prolonged exposure of the target cells to the therapeutic payload, maximizing the therapeutic effect. By combining the potential photothermal effect of rGO with the sustained release behavior of pEVs, GelAlg@rGO-pEV offers a synergistic approach to therapy, resulting in improved therapeutic outcomes.

**Fig. 7:**
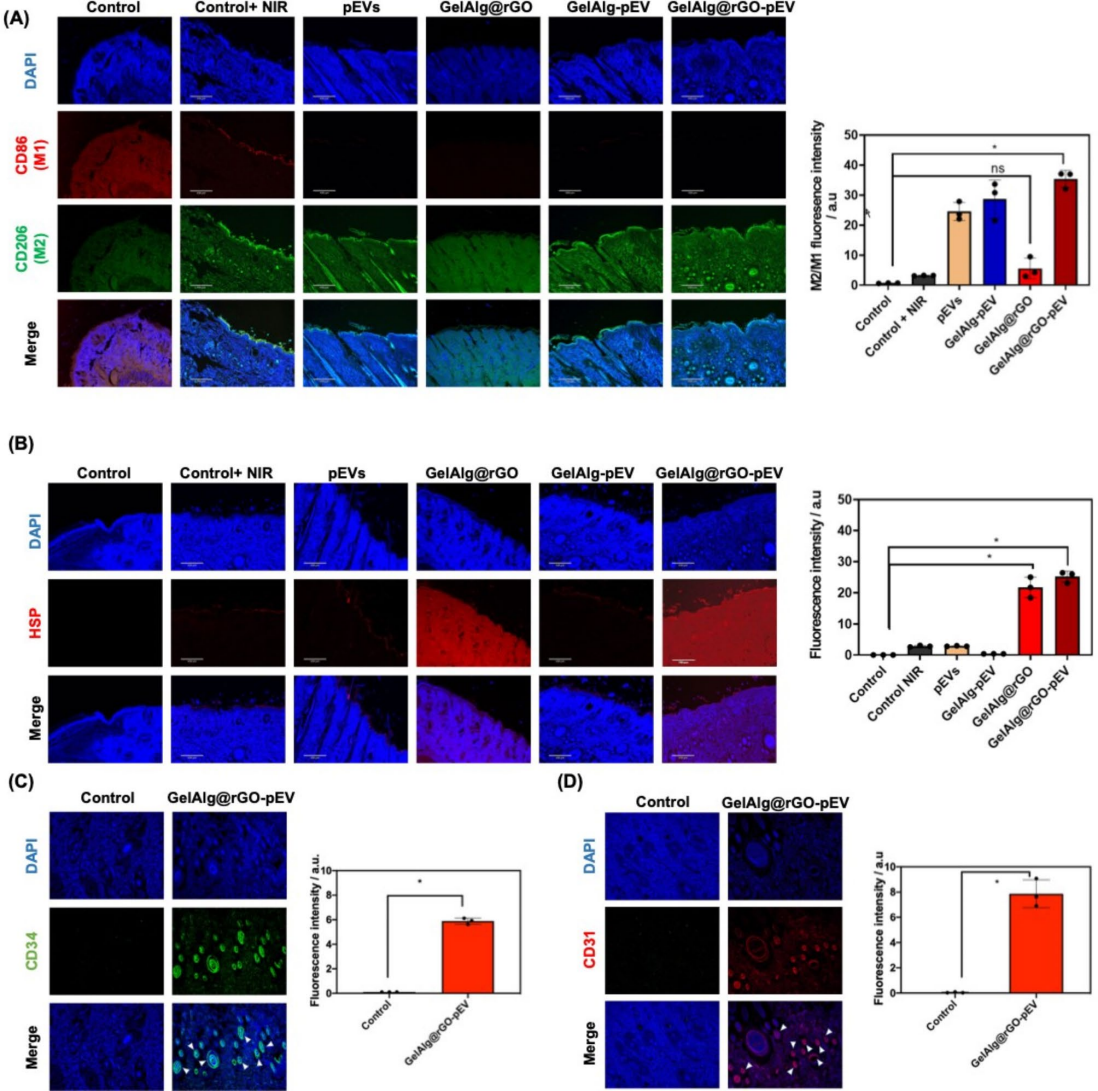
Macrophage polarization upon treatment with GelAlg@rGO-pEV: **(A)** M1/M2 macrophage and **(B)** heat shock protein (HSP) levels assessed in wounded skin organs by an immunofluorescence assay. **(C)** Follicle activation (CD34) and **(D)** Cluster of Differentiation 31 (CD31) angiogenesis levels were assessed in wounded skin organs by an immunofluorescence assay. The fluorescence intensity was quantified using ImageJ software. Data are represented as mean +/- SD, with n = 3 replicates; (ns > 0.05, *p < 0.05) White arrows indicate strong fluorescence signals

To examine whether pEVs-loaded GelAlg@rGO hydrogels can moderate the polarization of macrophages in diabetic wounds, histological immunofluorescence of M1 macrophages via CD86 and M2 macrophages (CD206) was conducted (Fig. 7A and **Figure S2)**, and M2 macrophages via CD206-was performed. The gel shows a strong M2 macrophage response.

This wound bandage also showed improved formation of heat shock proteins (HSP) (Fig. 7B and **Figure S2)** compared to the PBS control groups The GelAlg@rGO-pEV with NIR irradiation group showed improvement in follicle activation and angiogenesis, as seen in the immunofluorescence staining data (Fig. 7 C, D). These results suggest that the administration of GelAlg@rGO-pEV hydrogels with NIR irradiation to diabetic wounds had a favorable impact on the diabetic wound-healing process by promoting cell proliferation, anti-inflammation, immunomodulation, follicle activation, and angiogenesis. The value of the photothermal-responsive GelAlg@rGO-pEV hydrogel in promoting diabetic wound healing can be attributed to its multifunctional properties, which improve the hostile environment of chronic diabetic wounds.

## Conclusion

In summary, it could be demonstrated that the integration of rGO into GelAlg positively influences the mechanical properties of the hydrogel and confers photothermal properties into the gel. These properties were found to increase drug release when the gels were actuated at 808 nm. The properties of the formed GelAlg@rGO were tailored to load them with pEVs, showing that pEVs-loaded gels have favorable macrophage polarization and ROS-scavenging abilities. It is suggested that these gels mitigate M1 macrophage polarization and promote M2 macrophage polarization, reinforcing the notion that platelet proteins naturally loaded within pEVs have anti-inflammatory effects and can inhibit the polarization of M1 macrophages by modulating various signaling pathways. In vitro cell migration experiments validated that pEVs-loaded GelAlg@rGO gels improve the wound healing rate. With NIR activation, these gels were shown to result in complete wound closure in vivo. Moreover, the photothermally responsive GelAlg@rGO-pEV hydrogel promoted HSP expression, related to the cellular protective signaling pathway during diabetic wound healing. This multimodal GelAlg@rGO-pEV hydrogel has promising potential as a biomaterial for sequential biotherapy of diabetic wounds. In the future, addressing key aspects such as scale-up, sterilization, and quality control specifications will be critical to facilitating the translation of GelAlg@rGO-pEV hydrogel for clinical use. The demonstrated possibility of using clinical-grade human platelet concentrates, collected by licensed blood establishments, as the source material for pEVs production [38, 39, 41] further enhances the translational development potential of such EV-based hydrogels.

### Electronic supplementary material

Below is the link to the electronic supplementary material.

Supplementary Material 1

## Data Availability

The datasets used in this study are available from the corresponding author upon reasonable request.

## Declarations

### Corresponding authors

Correspondence to Er-Yuan Chuang (eychunag@tmu.edu.tw).
