# Supplementary material for: Enhanced diabetic wound healing using platelet-derived extracellular vesicles and reduced graphene oxide in polymer-coordinated hydrogels
Source: J Nanobiotechnology. 2023 Sep 4;21:318. doi: 10.1186/s12951-023-02068-x (PMC10478311; doi:10.1186/s12951-023-02068-x)
Supplement: Supplementary file 1 — Supplementary Material 1 [file 12951_2023_2068_MOESM1_ESM.docx]

**Supporting information**

**Figure S1:** Photothermal effect and cell toxicity toward L929 of aqueous solutions of different reduced graphene oxide concentrations when illuminate at 808nm at 2 W cm^-2^

**Figure S2**: **Fluorescence examination of wounded skin after 7 days treatment with different structures under 808 nm irradiation at 2 W cm^-2^.** (a) ROS, (b) M1 and M2 and (c) HSP.
